# Mechanosensitive TRPV4 Channel-Induced Extracellular ATP Accumulation at the Acupoint Mediates Acupuncture Analgesia of Ankle Arthritis in Rats

**DOI:** 10.3390/life11060513

**Published:** 2021-05-31

**Authors:** Yawen Zheng, Weimin Zuo, Dan Shen, Kaiyu Cui, Meng Huang, Di Zhang, Xueyong Shen, Lina Wang

**Affiliations:** 1 Acupuncture and Moxibustion College, Shanghai University of Traditional Chinese Medicine, Shanghai 201203, China; zhengy2w3@163.com (Y.Z.); wmzuo0207@shutcm.edu.cn (W.Z.); sd_ivy@126.com (D.S.); kaiyucui1018@163.com (K.C.); sxy1@shutcm.edu.cn (X.S.); 2 Shanghai Research Center for Acupuncture and Meridians, Shanghai 201203, China; mhuang@srcam.org; 3 Shanghai Key Laboratory of Acupuncture Mechanism and Acupoint Function (14DZ2260500), Department of Aeronautics and Astronautics, Fudan University, Shanghai 200433, China; dizhang@fudan.edu.cn

**Keywords:** acupuncture analgesia, mechano-sensitivity, TRPV4, extracellular ATP, nucleotidases

## Abstract

(1) Background: Acupuncture (AP) is a safe and effective analgesic therapy. Understanding how fine needles trigger biological signals can help us optimize needling manipulation to improve its efficiency. Adenosine accumulation in treated acupoints is a vital related event. Here, we hypothesized that extracellular ATP (eATP) mobilization preceded adenosine accumulation, which involved local activation of mechanosensitive channels, especially TRPV4 protein. (2) Methods: AP was applied at the injured-side Zusanli acupoint (ST36) of acute ankle arthritis rats. Pain thresholds were assessed in injured-side hindpaws. eATP in microdialysate from the acupoints was determined by luminescence assay. (3) Results: AP analgesic effect was significantly suppressed by pre-injection of GdCl_3_ or ruthenium red in ST36, the wide-spectrum inhibitors of mechanosensitive channels, or by HC067047, a specific antagonist of TRPV4 channels. Microdialysate determination revealed a needling-induced transient eATP accumulation that was significantly decreased by pre-injection of HC067047. Additionally, preventing eATP hydrolysis by pre-injection of ARL67156, a non-specific inhibitor of ecto-ATPases, led to the increase in eATP levels and the abolishment of AP analgesic effect. (4) Conclusions: These observations indicate that needling-induced transient accumulation of eATP, due to the activation of mechanosensitive TRPV4 channels and the activities of ecto-ATPases, is involved in the trigger mechanism of AP analgesia.

## 1. Introduction

As a traditional Chinese medicine, acupuncture (AP) has been gradually accepted worldwide. AP treatment has a superior efficacy in pain, including chronic and acute pain, and peripheral and visceral pain [1]. Although, to date, considerable basic studies have been executed to explore the underlying mechanisms of AP analgesia [2], it is still not fully understood how the anti-nociceptive signals are initiated by fine needles at the acupoints. A better understanding of this issue will guide clinicians to optimize the AP manipulation to improve AP efficacy.

Acupoints are special sites at the body surface which receive a variety of physical stimuli to modulate functional disorders of inner organs. According to our previous work on rats, mechanical stimulation was the main physical stimulus applied on acupoints during manual AP [3]. Our previous tests in rats revealed that liftinginserting manipulation generated a force in the range of 240–280 mN, and twisting manipulation produced a torque in the range of 10–15 mN × mm^−1^ [4], which were transmitted to the wider and deeper space by subcutaneous collagen fibers twisting around the needles [5,6]. We also showed that subcutaneous mast cells were present at a higher density in acupoints and their degranulation was one of the triggering signals in the mechanism of needling acupuncture analgesia [3]. Additionally, our previous in vitro work demonstrated that the transient receptor potential vanilloid 4 channels were expressed on the human mast cell line HMC-1, together with mechanosensitive TRPV2 [7] and chloride [8] channels. Further in vivo study unveiled that TRPV2-knockout arthritis mice had reduced response to AP [9]. TRPV4 is a mechanosensitive (MS) protein, which appears in various tissues or cell types, for example, keratinocytes [10], skeletal muscles [11,12], nerve fibers [13]. All of them are the main structural elements of acupoints.

It has been evidenced that needling promotes extracellular adenosine (Ado) accumulation in treated acupoints of humans [14] and rodents [9,15], which activates A1 receptors on nerve endings to trigger anti-nociceptive effects [9,15]. ATP is the precursor of Ado. ATP is a stress-responsive molecule, whose non-lytic release could be mediated by MS channels [16], including TRPV4 [16,17]. In the present work, we presumed that mechanosensitive release of ATP at the acupoints during needling would precede the accumulation of its metabolite Ado.

This work aimed to explore whether MS channels, especially TRPV4, at the acupoints were involved in triggering AP analgesia via promoting the accumulation of extracellular ATP (eATP) in the interstitial space. To address this issue, we used an acute arthritis pain rat model to explore the role of TRPV4 channels in AP analgesic effect and local eATP levels. We found that the AP analgesic effect benefited from a transient accumulation of eATP that was mediated by TRPV4 activation and ecto-ATPase activity.

## 2. Materials and Methods

### 2.1. Preparation of Reagents

GdCl_3_ (Sinopharm Chemical Reagent Company, Beijing, China), ruthenium red (RuR) (Sigma-Aldrich, St. Louis, MO, USA), 6-N,N-Diethyl-β-γ-dibromomethylene-D-adenosine-5′-triphosphatetrisodium salthydrate (ARL67156) (Sigma-Aldrich, St. Louis, MO, USA) and apyrase (Sigma-Aldrich, St. Louis, MO, USA) were prepared with distilled water. 2-Methyl-1-[3-(4-morpholinyl)propyl]-5-phenyl-N-[3-(trifluoromethyl)phenyl]-1H-pyrrole-3-carboxamide (HC067047) (Tocris, Minneapolis, MN, USA) was dissolved in dimethyl sulfoxide (DMSO). All of these stock solutions were kept at −20 °C and diluted into working solutions to final concentrations when used. DMSO was kept at less than 0.1% in the final solution. Finally, 4% paraformaldehyde (PFA) (Sinopharm Chemical Reagent Company, Beijing, China) was prepared in a fume hood and stored at 4 °C.

### 2.2. Animals

Adult male SD rats weighing 200 ± 20 g were used. All rats were randomly grouped into control, CFA model and AP-treated groups. For the behavioral tests, each group comprised 4–9 rats. For RT-PCR determination, each group comprised 4 rats. Rats were housed in a temperature-controlled (22–24 °C) room with a 12/12 h light/dark cycle and ad libitum access to food and water.

### 2.3. Acute Adjuvant Arthritis Model

Acute ankle arthritis was established by injection of 50 μL complete Freund’s adjuvant (CFA) (Sigma-Aldrich, St. Louis, MO, USA) into the left ankle joint cavity after rats were anesthetized with 1.5% isoflurane (Shanghai Yuyan Instruments Company, Shanghai, China). Local swelling and behavioral disability appeared within 24 h.

### 2.4. Behavioral Tests

**Figure 1:**
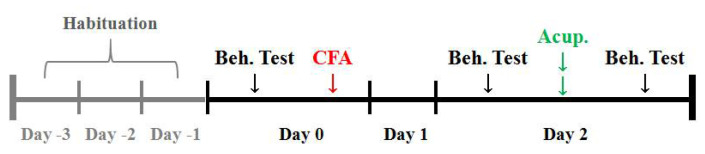
Timeline of the behavioral measurements. Before the tests, rats were allowed to acclimate for 3 days. CFA was administrated on day 0. AP was performed two days later. Pain thresholds were determined just before CFA injection (baseline) and before (modeling) and after treatment. Beh. Test: Behavioral test. Acup: Acupuncture treatment.

The timeline for pain threshold measurements is illustrated in Figure 1. Before the behavioral tests, rats were allowed to acclimate to the housing facilities for 3 days. Pain thresholds of the injured-side hind plantars were determined to reflect the pain levels. Thermal hyperalgesia was assessed using radiant heat (IITC336G, IITC Life Science, Woodland Hills, CA, USA), and the paw withdrawal latency (PWL) was defined as the time taken by rats to remove their hindpaws from the heat source. A cut-off time of 20 s was set to prevent potential tissue damage. Mechanical allodynia was evaluated by an electronic Von Frey anesthesiometer (IITC2450, IITC Life Science, Woodland Hills, CA, USA), and the paw withdrawal threshold (PWT) was defined as the force to push rats to withdraw their hindpaws. Each heat or force stimulus was applied 3 times at 10 min intervals at each time point and the average was taken as the threshold.

### 2.5. AP Treatments

AP treatments were performed on the affected side of conscious and unrestrained rats as described in our previous work [18]. According to our previous work, compared with other treatment sites along the sciatic nerve, including the lumbar Jiaji, Huantiao acupoint (GB30) and the main trunk of the sciatic nerve, the Zusanli acupoint (ST36) had the best anti-nociceptive effect on acute ankle arthritis rats [19]. Thus, ST36 was used in this study, which is located at the posterolateral side of knee joint, about 5 mm below the fibula capitulum. One treatment session of AP was executed on the 2nd day after CFA injection. A stainless steel needle (0.18 mm × 13 mm, Cloud Dragon Medical Equipment Company, Wujiang, China) was gently inserted to an approximate depth of 7 mm. The manipulation consisted of 30 s of lifting–thrusting and 30 s of twisting, and lasted for 20 min. To avoid confounding variables as much as possible, treatments on all rats were carried out by one person.

### 2.6. Acupoint/Intraplantar Injection

In order to intervene certain corresponding signals at the acupoints, GdCl_3_ (1 mM, 20 μL), RuR (0.5 mM, 20 μL), HC067047 (0.2 mM, 20 μL), ARL67156 (0.1 mM, 50 μL) or apyrase (10 U, 50 μL) were pre-injected into the left ST36 in layers 20 min prior to a 20 min AP treatment. In order to distinguish the different roles of TRPV4 channels at the acupoints and the pain sites, HC067047 (0.2 mM, 20 μL) or saline (20 μL) was pre-injected in the left plantar 40 min before behavioral tests.

### 2.7. ST36 Tissue Preparation

After the final behavioral tests, rats were euthanized with CO_2_. With the acupoints as the center, 5 × 5 × 5 mm specimens of tissues were taken out. For RT-PCR determination, acupoint tissues were immediately dissected, the skin and the muscle layers were separated, soaked in Trizol solution (Thermo Fisher Scientific, Whaltham, MA, USA) and stored in liquid nitrogen. For immunofluorescence staining, rats were first perfused with 0.9% saline (4 °C) transcardially and then with 4 % PFA solution (pH 7.40, 4 °C). The total perfusion volume of PFA for each rat was approximately 250 mL. Dissected ST36 tissues were soaked in the PFA solution for fixation (5 h, 4 °C), transferred to a 30 % sucrose solution for dehydration (72 h, 4 °C), embedded in optimal cutting temperature compound (OCT) and then frozen at −80 °C.

### 2.8. Immunofluorescence Staining on Cryosections

The samples in OCT were cut into 30 μm sections on a cryostat microtome (MEV, SLEE Medical GMBH, Mainz, Germany) and then the sections were thaw-mounted on positively charged covered glass slides (18 × 18 mm, Citogtest scientific, Haimen, China). Immunofluorescence staining was performed according to the protocol. In brief, before staining, the sections were rinsed with phosphate-buffered saline (PBS) and processed with goat blocking serum (10%, 50 μL) (Solarbio life sciences, Beijing, China) for 1 h (room temperature). Then, the slides were incubated with TPRV4 primary antibody (1:500, 50 μL) (cat # ab94868, Abcam, Cambridge, UK) overnight (4 °C). After washing with PBS, the slides were incubated in the corresponding secondary antibodies (1:500, 50 μL) (cat # ab150081, Abcam, Cambridge, UK) for 1 h (room temperature). Subsequently, the slides were counter-stained with DAPI reagent (Thermo Fisher Scientific, Waltham, MA, USA) for 10 min (room temperature) and then washed again. Finally, the sections were sealed by anti-fluorescence quencher (Biosharp, Hefei, China) and examined under a fluorescence microscope (Axio Obesever Z1, Zeiss, Oberkochen, Germany).

### 2.9. Microdialysis and Luminescence Analysis of eATP

Microdialysate collection: After rat was anesthetized with 1.5% isoflurane, a linear microdialysis probe with polyethersulfone membrane (cut-off 55 kDa; membrane length 10 mm) (CMA 31, CMA Microdialysis AB, Kista, Sweden) was implanted along the gap between the tibialis anterior and extensor digitorum longus. Subsequently, rat was restrained in the rat fixator. The dialysis solution was Hank’s balanced salt solution (HBSS) (Corning Cellgro, Manassas, VA, USA), which was controlled by syringe pumps (NE-1000, New Era Pump Systems, Farmingdale, NY, USA) and continuously perfused at a rate of 1 µL/min. After a 2.5 h recovery period, dialysate fractions were obtained before, during and after AP treatment.

eATP measurement: eATP levels in the fractions were quantified by luciferin-luciferase (L-L) assay (Sigma-Aldrich, St. Louis, MO, USA), and expressed as a molar concentration. Five-minute or 1 min microdialysate was transferred to each well of a 96-well plate. The same quantity (5 μL or 1 μL) of L-L mixture was added to each well and the light emission was measured immediately by a luminometer (Synergy Mx, BioTek, Winooski, VT, USA). In each experiment, calibration of luminescence versus ATP standards was performed with HBSS containing the corresponding compounds, which aimed to eliminate the interference of the reagents with the L-L assay.

### 2.10. Quantitative RT-PCR

Quantitative RT-PCR was performed as described in our previous work [20]. In brief, total mRNA was isolated from ST36 tissues. The primers for TRPV4 were: sense strand, 5′-AAGTGGCGTAAGTTCGGG-3′; antisense strand, 5′-TAAGGGTAGGGTGGCGTG-3′. The primers for NTPDase-1 were: sense strand, 5′-AGGAGCCTGAAGAGCTACC-3′; antisense strand, 5′-TGCCATAGAGTTCGCTTAC-3′. The primers for NTPDase-2 were: sense strand, 5′-GGCCAAAGGGCTACTCTACC-3′; antisense strand, 5′-GTTCCTGACAGGCTGACGAT-3′. The primers for NTPDase-3 were: sense strand, 5′-ACGGTTACAGCACCACCTTC-3′; antisense strand, 5′-ACAGCTGTGGGTCACCAGTT-3′. The relative abundance of specific mRNA was determined by the 2−ΔCt method, using β-actin as the internal standard.

### 2.11. Statistical Analysis

The data were analyzed and expressed as mean ± SE. The figures were prepared by GraphPad Prism 8.0 software (GraphPad Software, San Diego, CA, USA). Statistical significance was found using SPSS 24.0 (Chicago, IL, USA). Differences among multiple groups were tested with one-way ANOVA, followed by Fisher’s least significant difference (LSD) test. Where only two groups were compared, a two-sample *t*-test was used. When data were non-normally distributed, a *Mann-Whitney U test* was performed. *p* < 0.05 was considered statistically significant.

## 3. Results

### 3.1. AP Treatment at ST36 Relieved CFA-Induced Hypersensitivity in Hindpaws

**Figure 2:**
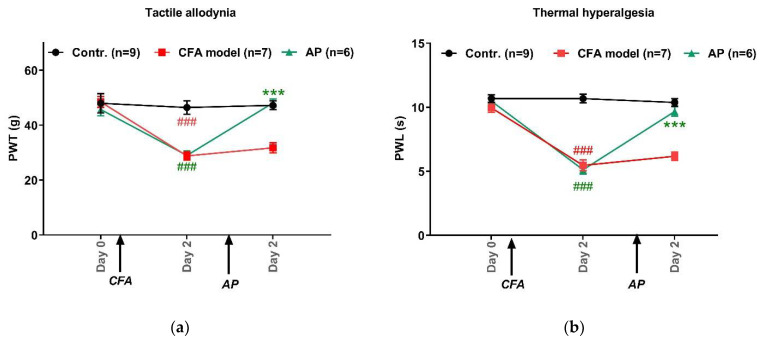
Effects of AP treatment on CFA-induced pain hypersensitivity (n = 6–9, mean ± SE). (**a**,**b**) Changes in tactile allodynia (PWT) and thermal hyperalgesia (PWL) of injured-side hindpaws, respectively. ### *p* < 0.001 vs. control. *** *p* < 0.001 vs. model.

Figure 2 shows the pain threshold changes in the injured hindpaws, including PWT and PWL. Baselines were similar for each group, 47.5 g ± 1.6 g for PWT (n = 6–9) and 10.4 s ± 0.2 s for PWL (n = 6–9). CFA-treated rats exhibited tactile allodynia (28.8 g ± 1.5 g, n = 7, *p* < 0.001 vs. control) and thermal hyperalgesia (5.5 s ± 0.4 s, n = 7, *p* < 0.001 vs. control). These pain rats clearly benefited from AP at ST36. Both PWT and PWL were nearly fully recovered to that of the control group (48.1 g ± 1.5 g, n = 6, *p* < 0.001 vs. model, for PWT; 9.7 s ± 0.4 s, n = 6, *p <* 0.001 vs. model, for PWL).

### 3.2. Inhibition of MS Channels at the Acupoints Partially Restrained AP Analgesic Effect

**Figure 3:**
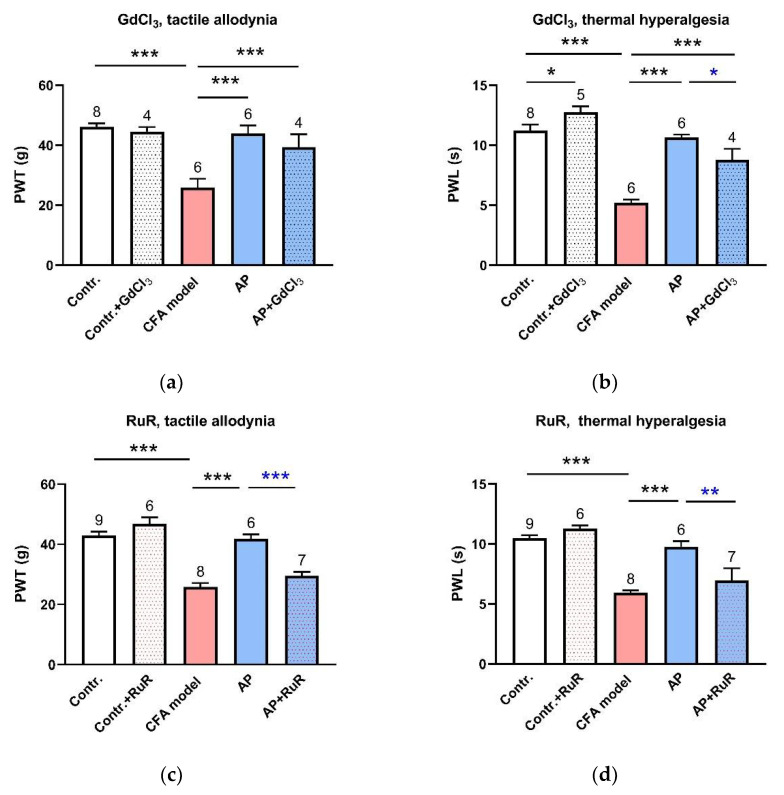
Involvement of MS channels at the acupoints in the AP analgesic mechanism (n = 4–9, mean ± SE). (**a**,**b**) Effects of pre-injection of GdCl_3_ (1 mM, 20 μL) in ST36 on AP-relieved PWT and PWL, respectively. (**c**,**d**) Effects of pre-injection of RuR (0.5 mM, 20 μL) in ST36 on AP-relieved PWT and PWL, respectively. Data illustrate the final behavioral tests. Numbers above each column denote sample size (n). * *p* < 0.05, ** *p* < 0.01, *** *p* < 0.001.

Both GdCl_3_ and RuR are the wide-spectrum antagonists of MS channels [21]. Pre-injection of either of them in ST36 did not affect the baseline PWT or PWL. However, GdCl_3_ (1 mM, 20 μL) partially suppressed AP-relieved PWL (n = 4, *p* < 0.05 vs. AP) but not PWT (Figure 3a,b). Compared with GdCl_3_, RuR (0.5 mM, 20 μL) had a stronger suppression effect. AP-induced anti-nociception was nearly abolished by RuR pre-injection (n = 6, *p* < 0.001 vs. AP, for PWT; *p* < 0.01 vs. AP, for PWL) (Figure 3c,d). These data suggest that local MS channels are activated by needling and mediate the AP analgesic effect.

### 3.3. TRPV4 Channels at the Acupoints Participated in the AP Analgesic Mechanism as Non-Nociceptive Protein

**Figure 4:**
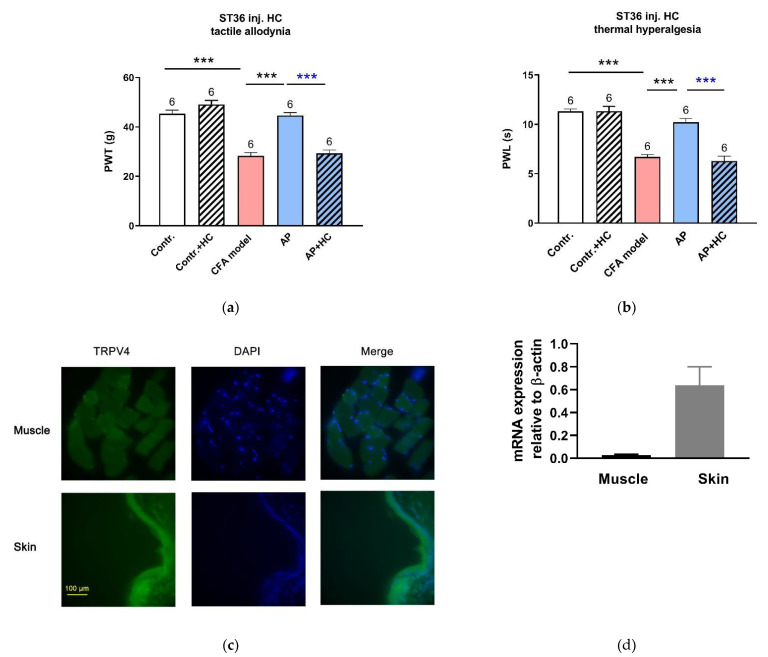
Involvement of TRPV4 channels at the acupoints in the AP analgesic mechanism (n = 6–9, mean ± SE). (**a**,**b**) Effects of HC067047 (0.2 mM, 20 μL) injection in ST36 on AP-relieved PWT and PWL, respectively. Data illustrate the final behavioral tests. Numbers above each column denote sample size (n). *** *p* < 0.001. (**c**) Immunofluorescence staining for TRPV4 in ST36. Both skeletal muscle and skin layers displayed a high TRPV4 expression (green). Nuclei were stained with DAPI (blue). Scale bar: 100 μm. (**d**) TRPV4 mRNA expression in muscle and skin layers excised from normal rats at ST36 (n = 4–5).

As for RuR, administration in ST36 with HC067047 (0.2 mM, 20 μL), a selective antagonist of TRPV4 channels, completely dampened the anti-nociceptive effect of AP on PWT (n = 6, *p* < 0.001 vs. AP) and PWL (n = 6, *p* < 0.001 vs. AP) (Figure 4a,b). Our immunofluorescence staining and RT-PCR assessment confirmed that TRPV4 was expressed in the acupoint tissues, especially in the skin layer (Figure 4c,d).

**Figure 5:**
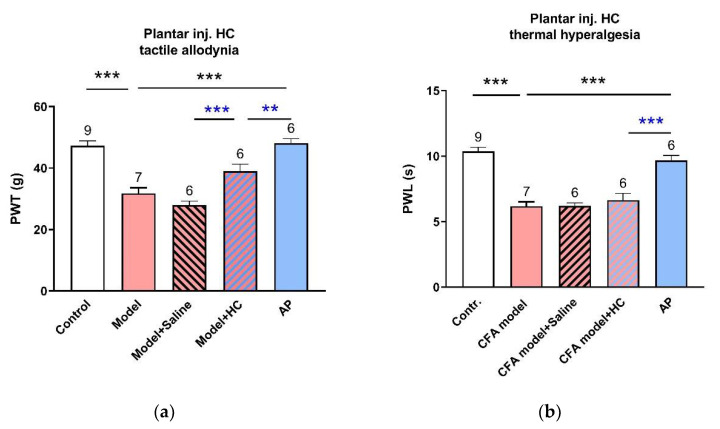
Effect of TRPV4 inhibition in injured plantar on CFA-induced pain hypersensation (n = 6–9, mean ± SE). (**a**,**b**) Effects of HC067047 (0.2 mM, 20 μL) pre-injection in the injured plantar on CFA-induced tactile allodynia and thermal hyperalgesia, respectively. Data illustrate the final behavioral tests. Numbers above each column denote sample size (n). ** *p* < 0.01, *** *p* < 0.001.

TRPV4 is commonly considered to be a pain-related protein [22,23]. In order to distinguish its different roles in the treated acupoints and pain sites, we further tested the responses of CFA-induced pain hypersensitivity to the inhibition of TRPV4 in the injured hindpaws (Figure 5a,b). Intraplantar injection of the same dose of HC067047 partially relieved the tactile allodynia (n = 6, *p* < 0.001 vs. CFA model+ saline injection), although it was not as effective as AP treatment (n = 6, *p* < 0.01 vs. AP) Thermal sensitivity remained unaffected.

Together, these findings imply that TRPV4 channels at the acupoints play a non-nociceptive role to mediate AP analgesia.

### 3.4. Needling-Induced eATP Transient Accumulation Mediated AP Analgesic Effect

**Figure 6:**
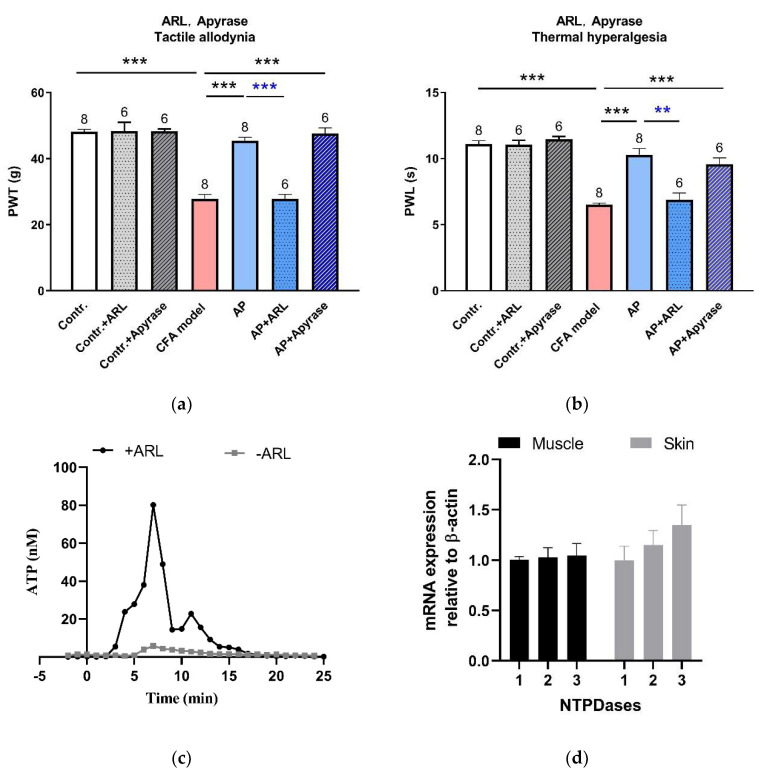
Involvement of eATP hydrolysis in the acupoints in the AP analgesic mechanism (mean ± SE). (**a**,**b**) Effects of ARL67156 (0.1 mM, 50 μL) and apyrase (10 U, 50 μL) on AP-relieved PWT and PWL, respectively (n = 6–8). Data illustrate the final behavioral tests. In a, data in the AP + ARL group were non-normally distributed, so the difference between the AP + ARL group and AP group was compared with *a Mann-Whitney U test*. ** *p* < 0.01, *** *p* < 0.001. Numbers above each column denote sample size (n). (**c**) Time-course of eATP changes during needling on CFA model rats in the absence and presence of ARL67156 (0.1 mM). Each sample contained 1 min microdialysate. These are representative traces from n = 6–7 recordings. (**d**) NTPDase1–3 mRNA expression in muscle and skin layers excised from normal rats at ST36 (n = 4).

As reported, needling-induced Ado accumulation in the interstitial space contributed to triggering the AP analgesic mechanism [9,15]. We hypothesized that such Ado accumulation resulted from the hydrolysis of eATP. To verify this conjecture, subsequently, we intervened the eATP hydrolysis at the acupoints (Figure 6a,b). Neither ARL67156 (0.1 mM, 50 μL), a non-specific inhibitor of ecto-ATPases, nor apyrase (10 U, 50 μL), a highly active ATP-diphosphohydrolase, affected PWT or PWL at baseline. ARL67156 abolished AP-relieved PWT (n = 6, *p* < 0.001 vs. AP) and PWL (n = 6, *p* < 0.001 vs. AP). Apyrase did not further improve the anti-nociception induced by AP. Determination of the microdialysate revealed that in CFA-treated rats, eATP transiently accumulated during needling, which was remarkably facilitated in the presence of ARL67156 (0.1 mM) in the perfusion HBSS (Figure 6c). These results indicate that ecto-ATPases were active at the acupoints, which hydrolyzed eATP and indirectly accelerated Ado accumulation via combining with ecto-AMPases. The nucleoside triphosphate diphosphohydrolase (NTPDase) family represents the major ecto-ATPases, which degrade ATP to AMP with intermediate formation of ADP [24]. Our RT-RCR uncovered that NTPDase1-3 mRNA was expressed in ST36, including skin and muscle tissues (Figure 6d). These findings imply that eATP hydrolysis, following its accumulation, is a further necessary step towards initiating AP anti-nociception.

### 3.5. TRPV4 Channels Mediated eATP Accumulation at the Acupoints during AP

**Figure 7:**
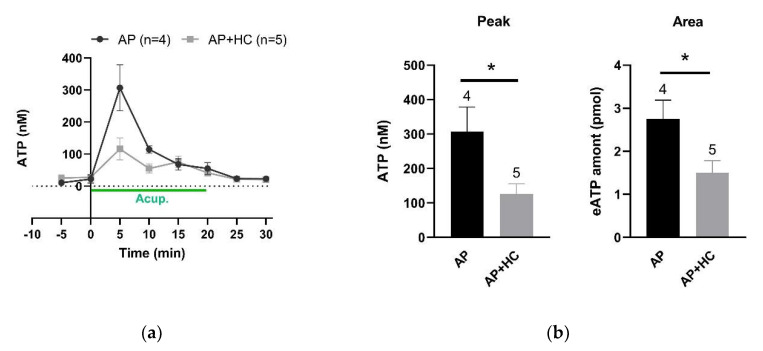
Effect of TRPV4 channel inhibition at the acupoints on needling-induced eATP accumulation (n = 4–5, mean ± SE). (**a**) Time-course of eATP changes during AP in the absence and presence of HC067047 (0.2 mM, 20 μL). Each sample contained 5 min microdialysate. (**b**) Comparison of eATP peaks and the total amounts, respectively, in the absence and presence of HC067047 (0.2 mM, 20 μL). Data based on (**a**). eATP amounts were calculated for the area under the curve from 0 to 20 min. * *p* < 0.05.

In CFA-treated rats, determination of microdialysis samples collected from ST36 unveiled that needling-triggered transient eATP accumulation was dramatically suppressed by local injection of HC067047 (0.2 mM, 20 μL) (Figure 7a). Further analysis of the peaks and the total amounts during the needling period revealed that blockage of TRPV4 channels significantly reduced local eATP levels (n = 4–5, *p <* 0.05) (Figure 7b). These results together with the above findings suggest that activation of TRPV4 channels at the acupoints contributes to AP analgesia via promoting eATP accumulation.

## 4. Discussion

### 4.1. AP Treatments Exerted Immediate Analgesic Effect on Acute Ankle Arthritis-Induced Pain Hypersensitivity

Both the current work and other similar studies [9,15] confirmed that AP treatments had an immediate anti-nociceptive effect (Figure 2), suggesting that neurophysiological mechanisms were involved. The hindpaw and ST36 are innervated by the tibial nerve and peroneal nerve, respectively. Both of them are the branches of the sciatic nerve that is the peripheral fiber trunk of lumbar 4–6 dorsal root ganglia. Hence, in this work, ST36 had the same spinal segmental innervation as the injured hindpaw. According to the principle of spinal segmental innervations, signals from acupoints and pain sites will interact in the spinal cord dorsal horn, which interferes with the transmission of noxious signals to reach the upper spinal cord level [2]. In addition, AP has a non-specific systemic effect, which is mainly attributed to the involvement of multiple brain areas and various neurochemical substrates [2,25]. For example, our previous work showed that CFA-induced ipsilateral arthritis resulted in bilateral pain hypersensitivity, which could be relieved by AP at the injured-side ST36 [26].

### 4.2. The Mechanism of AP Analgesia Involved the Activation of Local Mechanosensitive TRPV4 Channels

An acupoint is a three-dimensional structure, which contains some MS tissues or cells, for example, keratinocytes, skeletal muscles and microvessels. All of them could be activated by AP. Our previous work revealed that mechanosensitive TRPV2 [7] and mechanosensitive chloride channels [27] were expressed in mast cells. In vivo studies unveiled that the presence of this kind of cell at the acupoints and their degranulation contributed to the AP analgesic effect [3], and TRPV2 knockout arthritis mice had reduced response to AP [9]. The present work showed that blockage of MS channels at the acupoints with GdCl_3_ (Figure 3a,b) and RuR (Figure 3c,d) partially diminished and even nearly abolished the AP analgesic effect, respectively. RuR is not only a wide spectrum inhibitor of MS channels [21], but also a non-specific antagonist of TRPV channels, including TRPV4 [28]. Subsequently, specific inhibition of TRPV4 at the acupoints showed a similar effect to RuR (Figure 4a,b), indicating that activation of TRPV4 channels at the acupoints contributed to the AP analgesic effect. However, TRPV4 channels at the pain site (injured paw) contributed to tactile allodynia, because intraplantar HC067047 partially relieved PWT (Figure 5a). This finding was consistent with other reports, in which TRPV4 channels were considered as a pain sensor [22,23] to maintain mechanical allodynia via its mechanosensitivity [29]. Thus, our findings demonstrated a novel role of this protein in the pain-related field. There are various types of mechanosensors in the human body. Besides TRPV4, we also found the role of local transient receptor potential ankyrin 1 (TRPA1) and Piezo channels in acupuncture analgesia. They showed similar effects to TRPV4 (data not shown). The three differ in activation thresholds of mechanical stimuli. TRPV4 and TRPA1 channels are activated by a high threshold, but Piezo by light touch [30]. We are trying to figure out the different mechanisms among them underlying ATP release at the cellular level.

### 4.3. Needling-Induced Local eATP Accumulation Via Activation of TRPV4 Channels

When using high-performance liquid chromatography with a temporal resolution of 30 min, a small needling-mediated eATP elevation has been observed in mice [15] but not in human subjects [14]. Using the L-L assay with a comparatively higher temporal resolution of 5 min or 1 min, the current work recorded an obvious transient accumulation of eATP induced by needling at rat acupoints, which was dramatically reduced by local inhibition of TRPV4 channels (Figure 7), suggesting that AP-induced eATP accumulation was mediated by activation of TRPV4 channels. TRPV4-mediated ATP release in response to mechanical stimulation has been found in some cell types, including human epidermal keratinocytes [10], mouse primary urothelial cells [31] and human odontoblast-like cells [17]. TRPV4 channels have been reported to be expressed in keratinocytes [10], skeletal muscles [11,12] and mast cells [7], which was confirmed by the current work (Figure 4c) and our previous work. Additionally, nerve fibers also express TRPV4 channels [13]. All of them are the main structural elements of acupoints.

TRPV4 is a Ca^2+^-permeable, polymodally gated ion channel, which causes Ca^2+^ influx when activated [32]. Mechanosensitive ATP release correlates tightly with [Ca^2+^]*_i_* elevation [8,33]. TRPV4-induced [Ca^2+^]*_i_* increase is reported to mediate ATP release from airway smooth muscle of guinea pigs [34] and mouse esophageal keratinocytes [35]. Besides intracellular Ca^2+^ mobilization, pannexin channels are another element involved in TRPV4-mediated ATP release [36]. Whether these are the underlying mechanisms for TRPV4-mediated eATP accumulation in acupoints still needs further exploration.

### 4.4. Local eATP Hydrolysis was Necessary for AP Analgesia

In the present work, behavioral tests revealed that inhibition of eATP hydrolysis with ARL67156 at the acupoints completely canceled out the AP analgesic effect (Figure 6a,b), suggesting that eATP hydrolysis was a necessary step to initiate the analgesic mechanism. Our microdialysate determination confirmed the presence of highly active ecto-nucleotidases at the acupoints (Figure 6c). Various cell-attached or soluble ecto-nucleotidases precisely control eATP levels. Among them, the NTPDase family represents the major nucleotide-hydrolyzing enzymes, which degrade ATP to AMP with intermediate formation of ADP [24]. ARL67156 is a non-specific inhibitor of NTPDase-1, NTPDase-3 and nucleotide pyrophosphatase/phosphodiesterase 1 (NPP1) [37]. Our RT-PCR results revealed the expression of NTPDase1-3 mRNA at the acupoints (Figure 6d). Promoting eATP hydrolysis by apyrase did not further improve the anti-nociceptive effect of AP (Figure 6a,b), which might be due to the ceiling effect of AP.

Behavioral evidence in mice has shown that the nucleotidases prostatic acid phosphatase (PAP) [38] and ecto-5′-nucleotidase (Nt5e) [39,40], hydrolyzing ATP and AMP into Ado, respectively, have anti-nociceptive effects at the spinal cord level via promoting Ado production. Injection of PAP into the Weizhong acupoint (BL40) has an analgesic effect similar to AP treatment, which lasts up to six days following a single injection [41]. To date, despite the lack of behavioral evidence to support the anti-nociceptive effect of NTPDases, histomorphometric and histochemical studies have shown that NTPDase-3 is mainly expressed on nociceptive-associated small and medium neurons in sensory ganglia, and co-localized with markers of nociceptors [42,43]. In the skin, Langerhans cells express NTPDase-1 [44] and keratinocytes express NTPDase-3 [43] and Nt5e [45]. Together with our results above, we believe that nucleotidases are involved in the trigger mechanism of AP analgesia via facilitating eATP hydrolyzation into Ado.

## 5. Conclusions

At the acupoints, needling-induced transient accumulation of eATP, due to the activation of mechanosensitive TRPV4 channels and the activity of ecto-ATPase, mediates the trigger signals in the AP analgesic mechanism.

## Data Availability

Data are available from the corresponding author upon specific request.

## Abbreviations

Ado	Adenosine
AP	Acupuncture
ARL67156	6-N,N-Diethyl-β-γ-dibromomethylene-D-adenosine-5′-triphosphate
CFA	Complete Freund’s adjuvant
DMSO	Dimethyl sulfoxide
eATP	Extracellular ATP
HBSS	Hank’s balanced salt solution
HC067047	(2-Methyl-1-[3-(4-morpholinyl)propyl]-5-phenyl-N-[3-(trifluoromethyl)phenyl]-1H-pyrrole-3-carboxamide
L-L assay	Luciferin–luciferase assay
MS	Mechanosensitive
Nt5e	Ecto-5′-nucleotidase
NTPDases	Nucleoside triphosphate diphosphohydrolases
OCT	Optimal cutting temperature compound
PAP	Prostatic acid phosphates
PBS	Phosphate-buffered saline
PFA	Paraformaldehyde
PWL	Paw withdraw latency
PWT	Paw withdraw threshold
RuR	Ruthenium red
TRPA1	Transient receptor potential ankyrin 1
TRPV4	Transient receptor potential vanilloid 4
